# Helminth Antigens Enable CpG-Activated Dendritic Cells to Inhibit the Symptoms of Collagen-induced Arthritis through Foxp3+ Regulatory T Cells

**DOI:** 10.1371/journal.pone.0040356

**Published:** 2012-07-25

**Authors:** Franco Carranza, Cristian Roberto Falcón, Nicolás Nuñez, Carolina Knubel, Silvia Graciela Correa, Ismael Bianco, Mariana Maccioni, Ricardo Fretes, María Fernanda Triquell, Claudia Cristina Motrán, Laura Cervi

**Affiliations:** 1 Departamento de Bioquímica Clínica, CIBICI-CONICET, Facultad de Ciencias Químicas, Universidad Nacional de Córdoba, Córdoba, Argentina; 2 Centro de Excelencia en Productos y Procesos de la Provincia de Córdoba (CEPROCOR), Agencia Córdoba Ciencia, CONICET, Córdoba, Argentina; 3 Biología Celular, Histología y Embriología, Facultad de Ciencias Médicas, Universidad Nacional de Córdoba, Córdoba, Argentina; University of Southern California, United States of America

## Abstract

Dendritic cells (DC) have the potential to control the outcome of autoimmunity by modulating the immune response. In this study, we tested the ability of *Fasciola hepatica* total extract (TE) to induce tolerogenic properties in CpG-ODN (CpG) maturated DC, to then evaluate the therapeutic potential of these cells to diminish the inflammatory response in collagen induced arthritis (CIA). DBA/1J mice were injected with TE plus CpG treated DC (T/C-DC) pulsed with bovine collagen II (CII) between two immunizations with CII and clinical scores CIA were determined. The levels of CII-specific IgG2 and IgG1 in sera, the histological analyses in the joints, the cytokine profile in the draining lymph node (DLN) cells and in the joints, and the number, and functionality of CD4+CD25+Foxp3+ T cells (Treg) were evaluated. Vaccination of mice with CII pulsed T/C-DC diminished the severity and incidence of CIA symptoms and the production of the inflammatory cytokine, while induced the production of anti-inflammatory cytokines. The therapeutic effect was mediated by Treg cells, since the adoptive transfer of CD4+CD25+ T cells, inhibited the inflammatory symptoms in CIA. The *in vitro* blockage of TGF-β in cultures of DLN cells plus CII pulsed T/C-DC inhibited the expansion of Treg cells. Vaccination with CII pulsed T/C-DC seems to be a very efficient approach to diminish exacerbated immune response in CIA, by inducing the development of Treg cells, and it is therefore an interesting candidate for a cell-based therapy for rheumatoid arthritis (RA).

## Introduction

RA is an autoimmune disease that affects 1–2% of the population world wide and is caused by the loss of immunological self-tolerance leading to infiltration of the joint synovium by activated inflammatory cells, synovial hyperplasia, neoangiogenesis and the progressive destruction of cartilage and bone [1]. During the progression of the disease, Th1 and Th17 cells enter the joint tissues, releasing proinflammatory cytokines and chemokines which promote macrophage and neutrophil infiltration and activation [2]. Different therapeutic approaches to prevent the activation of inflammation have been developed for the treatment of RA. However, conventional treatments for autoimmune diseases are mainly immune suppressants, which have a variety of adverse effects and do not inhibit the inflammatory process in a specific manner [3].

DC are the most potent antigen presenting cells, which can be manipulated not only to activate lymphocytes, but also to induce T cell-tolerance to specific antigens, thereby minimizing autoimmune reactions [4]. Both immature and semi-mature DC have been associated with an induction of tolerance through the generation of regulatory T cells, the induction of apoptosis or the anergy of autoreactive effector cells [5]. In this way, DC can be used to induce tolerance *in vivo*. In animal models, manipulation of immature DC through different strategies have shown to be an effective methodology to inhibit the exacerbated immune responses in autoimmune diseases [6]. Among various stimuli, products from helminth parasites, after being recognized by innate immune cells, are capable of inducing several changes, such as the down-regulation of proinflammatory cytokines in TLR ligand maturated DC, the alternative activation of macrophages, the IL-10 production by Treg and B cells, and the production of IL-4 by basophils [7], [8]. Accordingly, in several experimental models, helminth infections have been able to suppress the inflammation through the down-regulation of the pathogenic T helper subsets (Th1, Th17, and allergic Th2) [7], [9], [10]. In addition, infection with *Fasciola hepatica* induces the suppression of immune responses to autoantigens and attenuates the clinical signs of experimental autoimmune encephalomyelitis [11].

In a recent work, we demonstrated that excretory secretory products from the helminth parasite *F. hepatica* have the ability to drive Th2 and Treg cells differentiation [12]. Furthermore, we have shown that *Fasciola hepatica* total extract (TE) is able to modulate LPS-induced DC maturation by decreasing pro-inflammatory cytokines and increasing IL-10 production [13]. To optimize the generation of tolerogenic DC, herein we explored whether the activation of DC with TE together with different TLR ligands could improve the tolerogenic properties of these cells. We found that DC simultaneously treated with TE and the TLR 9 ligand CpG (T/C-DC) exhibited an activation phenotype modulated by TE, characterized by high production of anti-inflammatory cytokines, moderated levels of pro-inflammatory cytokines and high costimulatory molecules and IDO expression. These T/C-DC pulsed with CII, promoted T cell tolerance, blunted Th1 and Th17 response and suppressed the inflammatory pathology in an experimental model of RA through mechanisms involving TGF-β induced Treg generation.

## Materials and Methods

### Animals

DBA/1J mice were purchased from Jackson Laboratories (Bar Harbor, ME). All mice were housed in the animal facility of the Department of Clinical Biochemistry of the Faculty of Chemical Science, National University of Córdoba, Córdoba, Argentina. Male mice, 8–12 weeks of age, were used in all the experiments. The Institutional Experimentation Animal Committee (authorization no. 15-01-44195) approved animal handling and experimental procedures.

### Antigens and Reagents

TE was obtained from mature flukes of infected bovine livers, as previously described [14]. Briefly, TE endotoxin contamination was removed by an endotoxin removing gel (Pierce Biotechnology, Rockford USA). LPS present in TE was determined by using the Endosafe Limulus Amebocyte Lysate test (Charles River Laboratories Wilmington, DE), and was similar to that of complete RPMI 1640 medium supplemented with 10% fetal calf serum (FCS; Gibco, Gran Island, NY), 50 µM 2-mercaptoethanol (Sigma-Aldrich, St Louis, MO) and 50 µg/ml Gentamicin (Gibco). CII was prepared as described [15]. CpG-ODN 1826 was purchased from OPERON (Huntsville, AL). LPS extracted from Escherichia coli (serotype 055:B5; Sigma-Aldrich). Zymosan was purchased from Sigma-Aldrich.

### DC Generation and Stimulation

DC were generated as previously described [16]. Briefly, bone marrow was collected from femurs of mice, and cells were seeded at 2×10^6^ cells in 10 ml of RPMI 1640 complete medium supplemented with 10% of supernatant from GM-CSF-producing J558 cells. Cells were fed on days 3 and 6 with complete RPMI medium containing GM-CSF. On day 8, harvested cells comprised 85% DC CD11c+. To stimulate DC, 2×10^5^ cells were treated with TE (80 µg/ml), CpG (10 µg/ml), LPS (1 µg/ml) or Zymosan 10 µg/ml) for 18 h. In all culture settings, cell viability was assessed by using an annexin V-FITC apoptosis detection kit (BD, Biosciences, San Diego, CA) with the dead-cell dye 7-AAD (Santa Cruz Biotechnology; San Diego, CA). The viability of DC after all treatments was about 75–80%**.**

### FACS Analysis of DC and Cytokine Production

The expression of surface molecules in DC was quantified by flow cytometry using FITC- or PE-conjugated Ab (CD11c, I-A/I-E, CD80, CD86, CD40) (BD PharMingen, San Diego, CA). Samples were collected using a cytometer FACSCanto™ II (BD San José, CA), and data analyzed by Flow Jo (Tree Star) software. The cytokine production in the supernatants of treated DC was measured by sandwich enzyme-linked immunosorbent assays (ELISA), using capture/biotinylated detection antibodies from BD Pharmingen.

### Western Blotting for IDO Expression and Activity

The IDO protein was analyzed in lysates from DC treated with medium, TE, CpG, or T/C for 24 h, by western blott. Then, immunoblotting was detected using polyclonal rabbit anti-IDO antibody (Santa Cruz Biotechnology, Santa Cruz, CA), the IDO protein was visualized using chemiluminescence substrate (GE Healthcare, Piscataway, NJ, USA). Anti-β-actin (Santa Cruz Biotechnology) was utilized as the loading control and analysis was carried out using the Gel-Pro analyzer 3.1. To measure IDO activity, DC were lysed by sonication for 10 seconds in an ice bath at a power of 100 W. Culture supernatant and cell lysates were used for the colorimetric assay of IDO activity as described by Kudo and Boyd [17].

### Induction of Arthritis and DC Therapy

DBA/1J mice were injected subcutaneously (s.c.) at the base of the tail with 150 µg of CII emulsified in an equal volume of CFA (Sigma Aldrich), supplemented with a suspension of *Mycobacterium tuberculosis* H37Ra (3 mg/ml). On day 21, the mice received a booster injection s.c. at the base of the tail with 150 µg of CII in IFA (Sigma-Aldrich). From day 30 (onset) to day 65, the swelling was scored in the limb joints by macroscopic examination. The arthritic severity in each paw was graded according to the established scoring system [18], 0 =  no swelling, 1 =  slight erythema, 2 =  slight swelling and erythema, 3 =  severe swelling 4 =  maximum swelling and deformity of limbs (maximum possible score 16 per animal). The mean arthritis score was the average of the scores for each group of mice from each day. 12 days after the first immunization, the mice were injected intraperitoneally (i.p) with 5–8×10^5^ DC treated previously with medium, TE, CpG or T/C and pulsed with 10 µg/ml of CII for 18 h. Control mice received PBS injections.

### Antibody Response to CII and Cytokine Production in DLN and Synovia

Blood samples were collected on day 35 of onset in differentially treated DC recipient CIA mice, and serum levels of CII and *M. tuberculosis* specific IgG2a and IgG1 were measured by sandwich ELISA as described before [19]. DLN were isolated from differentially treated DC recipient mice on day 10 of onset, and the cells were restimulated with CII (20 µg/ml) for 48 h. Levels of IL-17, IFN-γ, TGF-β and IL-10 were detected in supernatants of DLN cultures, and IL-17 and IFN-γ in joint protein extracts were measured by ELISA.

**Figure 1:**
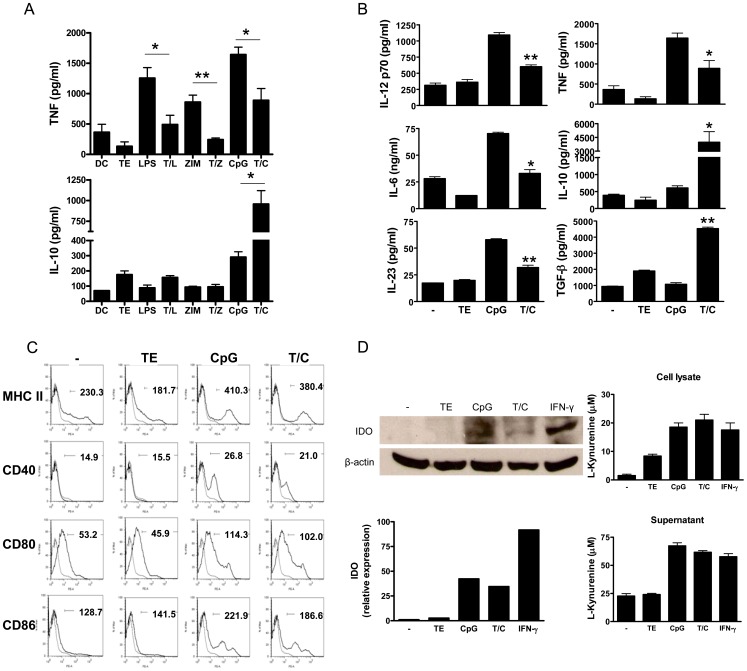
Cytokine production, phenotype and IDO expression by differentially-maturated DC. A) The DC were generated from DBA/1J mice and cultured for 18 h with LPS, Zymosan or CpG in presence or absence of TE. IL-10 and TNF were detected by ELISA. *P<0.05, **P<0.005 B) DC were cultured for 18 h with medium, TE, CpG and T/C. Supernatants were tested for IL-12p70, TNF, IL-6, IL-10, IL-23, TGF-β by ELISA. *P<0.05, **P<0.005 in IL-12p70, TNF, IL-6 and IL-23 versus CpG group, and *P<0.05, **P<0.005 in IL-10 and TGF-β versus the rest of the groups. C) DC were stimulated as described above and the expression of MHCII and costimulatory molecules (thick lines) was performed by FACS gating the cells on the basis of CD11c^+^ cells. Thin lines show isotype control and values represent the mean fluorescent intensity (MFI). D) Western blot analysis for IDO detection in whole lysates from DC treated as decribed above, using anti-β-actin Ab as the loading control. Data obtained were analyzed by scanning densitometry and were normalized according to the ratio IDO/β-actin expression and these values were compared to medium treated-DC (left panels). L-kynurenine concentration (IDO activity), was determined by a colorimetric assay in lysates or supernatants (right panels) respectively from DC treated as described above. Data shown are representative of two (D) or three (A, B and C) independent experiments. Bars in A, B and D are the means ± SD of triplicate wells per group.

### Suppressive Activity of Treg Cells from Mice Immunized with T/C-DC

To determine the percentage of Treg induced by vaccination with differentially treated DC and the IL-10 cell producers in DLN of animals immunized with T/C-DC, DLN cells were removed on day 10 of onset from animals treated as described above. Cells were stained with FITC–conjugated anti-CD4 mAb, PE- or APC-conjugated anti-CD25 mAb (clone 7D4 and PC61 respectively), FITC-conjugated anti B220 APC-Cy7-conjugated anti F4/80 (BD-Pharmingen). For intracellular IL-10 and Foxp3 staining, DLN cells were cultured for 5 hours with PMA (10 ng/ml) and ionomycin (1 mg/ml; Sigma-Aldrich) and brefeldin A (10 mg/ml; Sigma-Aldrich) was added for the last 4 hours of cell culture. The cells were stained for PE- or APC-conjugated anti-Foxp3 (clone FJK-16S, e-Bioscience) and PE-conjugated anti IL-10 antibody (BD-Pharmingen) using Foxp3 Fixation/Permeabilization. Concentrate and diluent and permeabilization buffer 10× (e-Bioscience) and analyzed by FACS. To test the TGF-β and IDO involvement in the expansion of Treg cells, co-cultures of DLN cells (2×10^5^/well) from CII/CFA immunized mice and CII pulsed T/C-DC (4×10^4^/well) were treated with anti-TGF-β (10 µg/ml**,** R&D Systems, MN) or the IDO inhibitor 1-methyl-D-tryptophan (1-MT, 200 µM, Sigma-Aldrich) and CII restimulated for 5 days. The percentage of Treg cells was analyzed by FACS.

**Figure 2:**
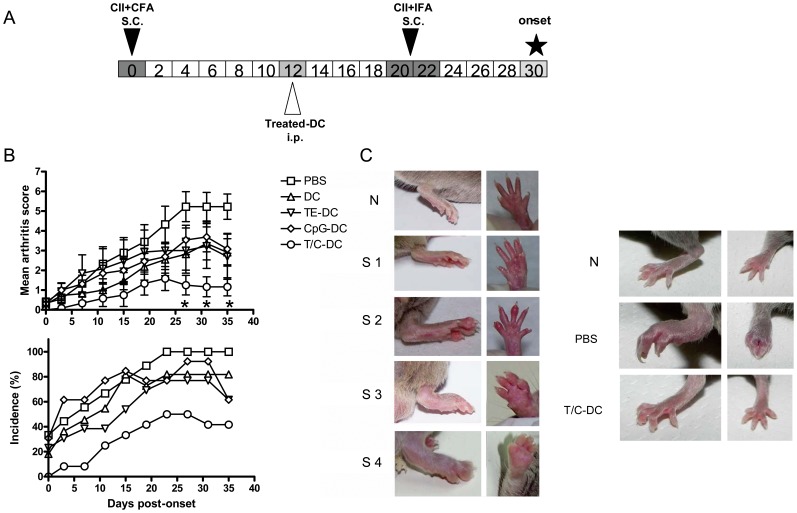
Diminished severity of CIA after treatment with CII pulsed T/C-DC. A) Scheme representing the experimental protocol followed, which is described in material and methods. B) The arthritis severity and incidence were assessed by clinical scoring from 0 to 35 days of onset in PBS (control), or in medium, TE, CpG and T/C-treated DC recipient mice. Values in arthritis score are the mean ± SEM of 12–15 mice per group and the incidence shows data compiled from 3 independent experiments, *P<0.05 for T/C-DC versus PBS on days 27, 31 and 35. C) Photographs show representative examples of the paw swelling in mice with different clinical scores (left panel), and normal, PBS and T/C-DC groups (right panel). N: normal, S: score.

To evaluate the effect TGF-β blockage in IL-10 production, DLN cells (2×10^5^/well) were removed on day 7 from CII/CFA immunized mice, and then co-cultured with CII pulsed differentially treated DC (4×10^4^/well) in the presence or absence of anti-TGF-β (10 µg/ml, R&D Systems, MN). The cells were restimulated with CII for 72 h and IL-10 concentration was measured by ELISA.

To determine the suppressive capacity of Treg cells, DLN cells from CII pulsed T/C-DC treated mice were obtained on day 7 of onset, and CD4+CD25+ and CD4+CD25- cell populations were sorted by using flow cytometer FACSAria™ II (BD San José, CA) with a purity of 96.5% and 98.5% respectively (Supplementary Figure S2 A).

Then, both populations were injected intravenously (3×10^5^ cells/mouse) on day 15 after the first immunization with CII/CFA in recipient mice. Joints were removed, fixed and embedded in paraffin, sectioned and stained with HE and analyzed with a Zeiss Axioscop microscope and the Axiovision 4.8.2.0 software (Carl Zeiss Vision, Germany).

**Figure 3:**
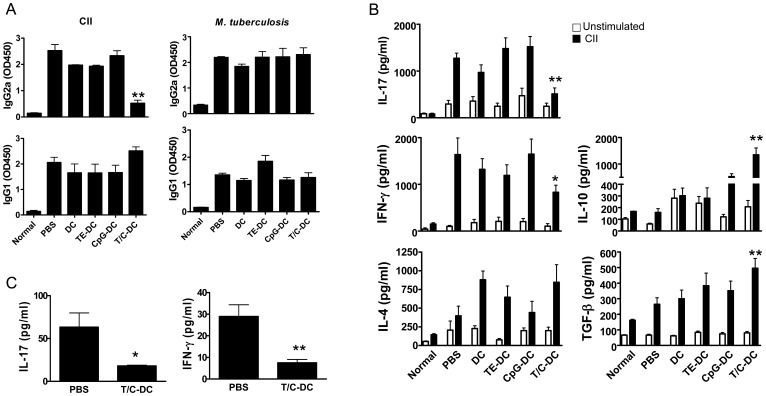
Decreased inflammatory responses after treatment with CII pulsed T/C-DC in CIA mice. A) IgG2a and IgG1 production against CII and *Mycobateria tuberculosis* were measured by ELISA in the sera from differentially treated DC recipient mice on day 35 of onset. Data are representative of two independent experiments. Values show the mean ± SD of optical density at 450 nm (OD450) of 5 mice per group. B) IL-17, IFN-γ, IL-4, IL-10 and TGF-β levels were detected by ELISA in the supernatant of cultures of DLN cells from mice on day 10 of onset stimulated with or without CII for 48 h. C) IL-17 and IFN-γ were measured by ELISA in joint homogenate. Data are representative of three independent experiments. Values are the mean ± SD. *P<0.05, **P<0.005 versus PBS group in A, B and C.

For *in vivo* CD25+ T cell depletion, DBA/1J mice were injected i.p. with 500 µg/mouse/day of purified anti-CD25 Ab, clone PC61, or a control isotype Ab, clone GL113 on days −4, −2, 2, and 4 of the injection with CII pulsed T/C-DC. We thank Fred D. Finkelman (University of Cincinnati Medical Center, Cincinnati, OH) for provision of PC61 and GL113 cell lines. The extent of CD25+ cell depletion in DLN was determined by FACS analyses stained with PE-anti rat anti mouse CD25 (BD, Pharmingen, clone 7D4) in normal mice after three injections with 500 µg/mouse/day of PC61.

**Figure 4:**
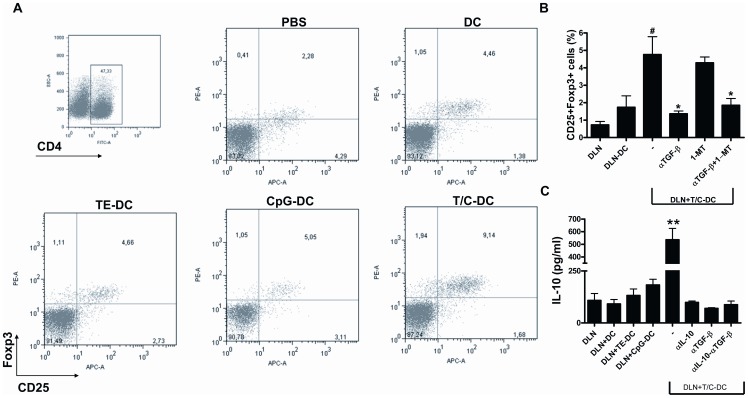
Enrichment of CD4+CD25+Foxp3+ in DLN cells by CII pulsed T/C-DC treatment. A) Cells from DLN of differentially-treated DC recipient mice were collected on day 7 of onset and stained with fluorescent antibodies for CD4, CD25 and Foxp3. Plots show the percentage of CD25+Foxp3+ cells gated on CD4+ cells. B) Cells from DLN of CII immunized mice cultured with T/C-DC plus CII for 5 days in the presence of neutralizing antibodies against TGF-β or 1-MT. Cells were gated on CD4+ cells, and bars represent the percentage of CD25+Foxp3+ cells. #*P*<0.05 versus DLN+DC, *P<0.05 versus DLN+T/C-DC. C) DLN cells from CII immunized mice were cultured with CII pulsed differentially treated DC in the presence of anti TGF-β and IL-10 production was analyzed by ELISA. **P<0.005 versus DLN + T/C-DC αTGF-β. Data are representative of two independent experiments.

### Statistical Analysis

Differences in the clinical scores were analyzed with the parametric Tukey-Kramer (ANOVA) test, and the Student’s *t-*test was used for analysis of the ELISA data. To examine the percentage of Treg cells in the co-cultures of DLN and CII pulsed T/C-DC, the Newman-Keuls Test (ANOVA) was used. *P* values less than 0.05 with a 95% confidence interval were considered statistically significant.

## Results

### Generation of Tolerogenic DC by T/C Treatment

In recent years, different strategies have been used to explore the ability of tolerogenic DC to prevent or at least control autoimmune processes. The characteristics of tolerogenic DC include the ability to secrete high levels of anti-inflammatory cytokines such as IL-10 and TGF-β, to produce low levels of pro-inflammatory cytokines and to express IDO [5]. In a previous work, we demonstrated that TE modulated LPS-induced DC maturation, increases the amount of IL-10 and reduces IL-12p70 production [13]. Here, we studied the capacity of TE to modulate DC maturation induced by LPS, Zymosan or CpG, to produce IL-10 and TNF. Among the different TLR ligands, CpG in combination with TE induced the highest production of IL-10 and reduced the TNF production (Fig. 1 A). Based on these data, we explored whether T/C treatment could enable DC with additional tolerogeneic properties to be used to prevent an *in vivo* inflammatory response. To carry this out, DC from DBA/1J mice were treated with medium, TE, CpG or T/C and after 18 h we measured the cytokine production by ELISA and the expression of MHC class II, CD40, CD80 and CD86 molecules by FACS. T/C-DC produced significantly higher levels of IL-10 and TGF-β than the rest of the treatments. In addition T/C induced in DC lower levels of the pro-inflammatory cytokines IL-12p70, TNF, IL-6 and IL-23 compared with those produced by CpG-treated DC (Fig. 1 B). Besides, T/C-DC showed similar levels of MHCII, CD40, CD80 and CD86 expression compared with CpG treatment (Fig. 1 C). In addition, IDO protein was detected by Western blot of DC lysates. A 42-kDa band corresponding to IDO was up regulated in CpG- and T/C-treated DC compared to untreated DC (Fig. 1 D left pannels). Besides, the IDO activity was determined in cell lysates by the L-kynurenine concentration in culture supernatants from differentially treated DC. As shown in Fig. 1D right panels, CpG and T/C treatment both were able to induced IDO activity in DC. As expected, IDO expression and activity was induced in IFN-γ-treated DC (positive control) (Fig. 1 D).

**Figure 5:**
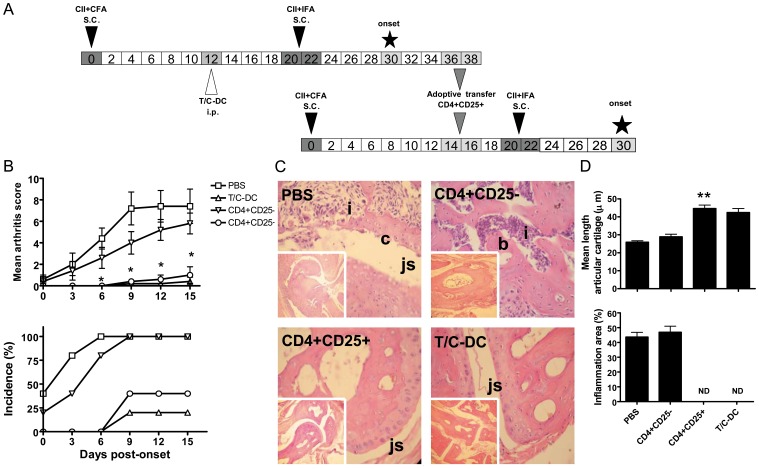
Decreased severity of CIA following adoptive transfer of CD4+CD25+ T cells induced by the CII pulsed T/C-DC treatment. A) Scheme representing the experimental protocol followed to induce and transfer CD4+CD25+ T cells, which is described in material and methods. B) Mice were injected with PBS, CII pulsed T/C-DC (5–8×10^5^/mouse), CD4+CD25-, CD4+CD25+ T cells, (3×10^5^/mouse), and the arthritis severity and incidence were assessed by clinical scoring during 15 days of onset. Values in arthritis score are the mean ± SEM of 5 mice per group, *P<0.05 for CD4+CD25+ versus PBS on days 6, 9, 12 and 15. C) Histopathological examination of representative joints of PBS, CD4+CD25−, CD4+CD25+ T cells or CII pulsed T/C-DC recipient mice on day 15 of onset, original magnifications 10× (small squares) and 40× (big squares). The pictures show: inflammatory cell infiltrates (i), bone erosion (b), cartilage erosion (c), joint space (js). D) Graphs representing the measurement of the mean length of the articular cartilage and inflammation area from 5 points in each sample. Values are the mean ± SD of 5 mice per group. **P<0.005 versus PBS on day 15 of onset.

**Figure 6:**
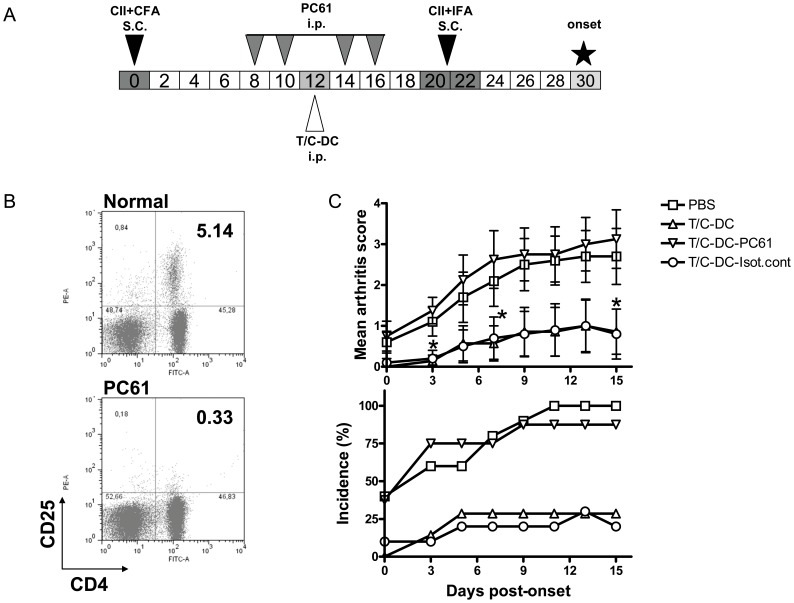
Depletion of CD25+ cells abrogated the ability of CII pulsed T/C-DC to inhibit the symptoms of CIA in mice. A) Mice were injected i.p. with four injections of 500 µg/mouse/day of anti-CD25 Ab (clone PC61) or the same amount of isotype control Ab (clone GL113), as shown in the scheme. B) Depletion of CD4+CD25+ T cells was checked in DLN by FACS with anti-CD4 and anti-CD25 Abs (clone 7D4). A representative staining pattern is shown. C) Mice were injected with PBS; CII pulsed T/C-DC, CII pulsed T/C-DC plus the treatment with anti-CD25 Ab or CII pulsed T/C-DC plus the treatment with anti-isotype control Ab. The arthritis score and incidence were assessed by clinical scoring during 15 days of onset. Values in arthritis score are the mean ± SEM of 7–10 mice per group and the incidence shows data compiled from 3 independent experiments, *P<0.05 for T/C-DC versus T/C-DC-PC61 on days 3, 7 and 15 of onset.

Taken together, these findings indicate that DC from DBA/1J mice stimulated with T/C displayed an activation phenotype modulated by TE with a moderate production of proinflammatory cytokines, high levels of anti-inflammatory cytokines and high co-stimulatory molecules and IDO expression.

### Decreased Severity of Experimental CIA Progression by Treatment with T/C-DC

Next, we investigated whether vaccination with CII pulsed T/C-DC could modulate the outcome of CIA. The presence of CII in DC cultures with the different stimuli, did not modify their maturation status and cytokine profiles (data not shown). The mice were vaccinated as described in Materials and Methods, following the scheme shown in Fig. 2 A. After CII pulsed T/C-DC treatment, the mice displayed lower arthritis score and incidence compared with the rest of the groups (Fig. 2 B), according to inflammation signals in the paws (Fig. 2 C, left panel), with the arthritic score being significantly different from the PBS (control group) from day 27 to 35 of onset (Fig. 2 B). Fig. 2 C (right panel), shows that the extension of the inflammation was much lower in the recipient mice of CII pulsed T/C-DC than in the PBS control group. Together, these data indicates that treatment with CII pulsed T/C-DC was able to ameliorate the clinical symptoms of CIA, thereby reducing the arthritis score and incidence.

### Reduced CII-specific IgG2a Production After Vaccination with CII Pulsed T/C-DC

As CII-specific Abs are crucial for disease induction and progression [20], we next evaluated whether CII-specific Ab responses were affected by vaccination with CII pulsed T/C-DC. The production of CII-specific IgG2a (Th1-associated IgG isotype) and IgG1 (Th2-associated IgG isotype) in the sera of mice was measured by ELISA. The production of CII specific IgG2a Ab in animals injected with CII pulsed T/C-DC was significantly diminished compared with that observed in PBS control mice, while IgG1 was unmodified (Fig. 3 A). The level of the IgG2a isotype was reduced in an antigen-specific way, since the production of Abs against an unrelated Ag (*Mycobacterium tuberculosis*) was unaffected compared to PBS control mice (Fig. 3 A). Taken together, these data indicate that the treatment with CII pulsed T/C-DC was able to control the specific humoral immune response associated with inflammatory response (IgG2a).

### Down-regulation of Proinflammatory Cytokine Production in CIA Mice After Treatment with CII Pulsed T/C-DC

We next investigated whether the reduction of the symptoms in CIA induced by CII pulsed T/C-DC could be related to changes in the CII-specific cellular immune response. To carry this out, DLN cells taken at day 10 of onset of CIA mice that had received differentially activated DC were restimulated with CII for 72 h, and the cytokines were measured in the supernatants by ELISA. Significantly lower levels of IL-17 and IFN-γ, but higher production of TGF-β and IL-10 were secreted by DLN cells from mice injected with CII pulsed T/C-DC compared to PBS injected mice, whereas IL-4 was not modified (Fig. 3 B). In a similar way, the proinflammatory cytokines IFN-γ and IL-17 were significantly reduced in the supernatants from a homogenate of the knee joint of mice injected with CII pulsed T/C-DC compared to the PBS group (Fig. 3 C). These results show that the vaccination with CII pulsed T/C-DC modulates the specific cellular response *in vivo*, decreasing the IL-17 and IFN-γ production in DLN as well as in the joints, and increasing IL-10 and TGF-β levels in DLN.

### Development of Treg Cells in CIA Mice After Immunization with CII Pulsed T/C-DC

It has been previously demonstrated that Treg cells induce protection against CIA by diminishing joint inflammation [21]. Therefore, we wondered whether the treatment with CII pulsed T/C-DC is capable of increasing the percentage of Foxp3 cells *in vivo.* Mice with CIA were immunized with differentially activated DC as described above, and on day 7 of onset, the DLN cells were removed and the cells were analysed for CD4 CD25 and Foxp3 expression by FACS. DLN cells from CII pulsed T/C-DC recipient mice showed a four-fold increase in the population of Treg cells compared to control mice (2.28% vs 9.14%) (Fig. 4 A), indicating that the vaccination with CII pulsed T/C-DC was able to induce or promote de proliferation of Treg cells. Given that TGF-β secretion and IDO expression by DC have been shown to be involved in the induction and expansion of Treg [22], [23], with these both factors being highly induced by the T/C treatment in DC, we investigated whether TGF-β blockage or the inhibition of IDO activity revert the increased of Treg. To carry this out, we co-cultured DLN cells from CII immunized mice together with CII pulsed T/C-DC in the presence or absence of anti-TGF-β Ab or 1-MT (a pharmacological IDO inhibitor). The presence of anti-TGF-β, resulted in the decrease in the percentage of Treg cell, whereas the addition of 1-MT has no effect on the number of these cells (Fig. 4 B). The combination of both inhibitors resulted in a similar reduction of the percentage of Treg cells than that observed in the presence of anti-TGF-β alone (Fig. 4 B).

Besides, the blockage of TGF-β in co-cultures of DLN cells from CII immunized mice together with CII pulsed T/C-DC inhibited IL-10 production compared to the level of this cytokine in the absence of the anti- TGF-β (Fig. 4 C). Accordingly, we found that the main source of IL-10 in the DLN from CII immunized and injected with CII pulsed T/C-DC, were neither B cells nor macrophages, but Foxp3+ cells population (Supplementary Figures S1 A, B and C).

Thus, we demonstrated that the ability of CII pulsed T/C-DC to develop Treg cells is dependent on TGF-β production and independent of IDO activity. Besides, IL-10 production was inhibited by TGF-β blockage, suggesting the involvement of Foxp3 cells in IL-10 production.

### Protection Against CIA by the Adoptive Transfer of CD4+CD25+ T Cells Induced by CII Pulsed T/C-DC Vaccination

The observed tolerogenic activity of CII pulsed T/C-DC and their ability to promote Treg cells development prompted us to investigate the relevance of the induced Treg cells in preventing inflammation and tissue damage. To address this, CD4+CD25+ T cells sorted from CII pulsed T/C-DC treated mice at day 7 post onset were transferred into CIA recipient mice, following the experimental scheme showed in Fig. 5 A. The percentage of Foxp3+ cells in CD4+CD25+ population was about 85% (Supplementary Figure S2 B). The analysis of the CIA symptoms in the recipient mice showed that the adoptive transfer of CD4+CD25+ cells significantly decreased the arthritis score and incidence from day 6 to 15 of onset, compared with the PBS group. In contrast, the transfer of CD4+CD25- T cells did not protect against CIA development (Fig. 5 B). The protective effect was further analyzed by histological examination. Joints of PBS or CD4+CD25- T cells treated mice were severely damaged with extensive accumulation of inflammatory cells occurring in the synovia, together with a reduction in the length of the articular cartilage, tissue destruction and erosion. In contrast, CII pulsed T/C-DC or CD4+CD25+ recipient mice showed a preserved architecture in the joints without apparent damage (Fig. 5 C and D). In order to further confirm the involvement of Treg cells in the suppression of the inflammatory symptoms, we depleted these cells by treating CII pulsed T/C-DC recipient mice with anti-CD25 mAb (PC61) as shown in the scheme (Fig. 6 A). Depletion with this antibody is known to eliminate 70% of Tregs [24]. We performed PC61 mAb injections during three days, resulting in more than 90% depletion of CD4+CD25+ T cells (Fig. 6 B). Depletion of CD25+ T cells in CII pulsed T/C-DC recipient mice significantly abrogated the suppressive effect of these cells on arthritis score and incidence (days 3, 7 and 15 of onset) compared to CII pulsed T/C-DC treatment (Fig. 6 B). Taken together, these results show that Treg cells induced by CII pulsed T/C-DC treatment were able to reduce the severity of the inflammation at a local level, since the structure of joints was highly preserved.

## Discussion

In this study, we explored a cell-based preclinical strategy for CIA using DC stimulated with helminth antigens plus a TLR ligand. Among different TLR ligands used in combination with TE, CpG was the most efficient stimulus to induce tolerogenic properties in DC. T/C-treated DC displayed an activation phenotype modulated by TE, as determined by the expression of surface markers as well as the cytokine production.

In recent years, has become apparent the ability of immature or semi-mature DC to induce tolerance [25], [26], [27]. Several agents such as TNF, IL-10, dexamethasone, vitamin D3 and vasoactive intestinal peptide have been reported to modulate the maturation of DC transforming these cells into immature or semi-mature phenotypes; thus affecting the outcome of the adaptive immune response by inducing tolerance through Treg cells generation or other protective mechanisms against autoimmune disease [28], [29], [30]. In this work TE modulates the CpG-induced activation of DC a nonconventional activation phenotype with tolerogenic properties.

In this work, the treatment of DBA/1J mice with CII pulsed T/C-DC reduced the severity and incidence on the CIA symptoms, and also prevented joint damage.

Our data revealed the increase of CD4+CD25+Foxp3+ T cells following vaccination with CII pulsed T/C-DC, which were capable of inhibiting the progression of CIA after transfer in recipient mice. Confirming these data, the depletion of CD25+ cells significantly abrogated the ability of CII pulsed T/C-DC to inhibit the symptoms in CIA mice. Several reports have shown that TGF-β and IDO are involved in the expansion and conversion of Treg cells [31], [32], [33], [34]. In this study, we have demonstrated the ability of T/C treatment to increase the production of these two factors in DC, suggesting their involvement in the mechanisms of Treg cells development. However, *in vitro* blockage of TGF-β but not of the IDO enzymatic activity, was capable of inhibiting the development of Treg cells. In agreement with these findings, it was demonstrated that autocrine or paracrine signaling through TGF-β induces in DC a long-term IDO-non enzymatic dependent effect that participate in both up-regulation of TGF-β secretion and in the generation and maintenance of Treg cells [35]. In contrast, the rapid (acute) IDO induction in DC by inflammatory stimulus like LPS (and possibly CpG) drive IDO-activity dependent effect that are, consequently, inhibited by 1-MT [35]. In line with this, we have observed that despite the high IDO expression and activity observed in CpG- treated DC, the immunization of CIA mice with these cells was unable to prevent the CIA symptoms. Overall these findings suggest that the TGF-β-IDO pathway described by Pallota *et al*. [35] could be a key factor in promoting the expansion or the novo generation of Treg cells [36].

Several mechanisms of action of Treg, such as inhibitory cytokines, cytotoxic molecules, modulators of cAMP and cytokine competition have all been demonstrated [37]. In this study, the high concentration of TGF-β in the supernatants of the DLN cells from CII pulsed T/C-DC recipient mice, suggests the potential involvement of this cytokine as an effector mechanism of Treg cells. Additionally, the high levels of IL-10 in DLN from CII pulsed T/C-DC recipient mice lead us to consider that signaling through IL-10/IL-10R *in vivo* could resulted in some mechanisms by which IL-10 act [38]. In this sense, according to our data the main source of IL-10 in CIA mice injected with CII pulsed T/C-DC was the Foxp3+ cells,. Confirming these data, the abrogation of TGF-β inhibited IL-10 production in DLN from CIA mice co-cultured with T/C-DC, probably due to a diminution in the generation of TGF-β-dependent Foxp3 cells producing IL-10.

Due to the ability of helminth Ags to polarize the T cell response toward the Th2 profile [39], which is known to be able to inhibit the Th1 and Th17 responses [40], [41], we reasoned that this mechanism could be involved in the inhibition of the inflammation in CIA mice. However, our data did not confirm this hypothesis, since neither a difference in IL-4 production in DLN nor in IgG1 production (Th2-associated IgG isotype) in the sera from differentially activated DC recipient mice were observed.

Despite some therapies being effective for RA, progression of the disease is still usually observed and some patients do not respond to therapy at all. Unlike conventional treatments for arthritis in which general immunosuppressants are used [26], treatment with CII pulsed T/C-DC revealed three main advantages: firstly, the reduction of the pathogenic IgG2a Ab was Ag specific, with this fact at preventing the compromise of the protective immunity as occurs from the risk of infections and malignancy in RA patients treated with immunosuppressive drugs. Secondly, the CII pulsed T/C-DC treatment was effective when administrated before the onset of clinical symptoms in CIA, which might have an application in individuals at risk of developing arthritis, such as in patients in the long preclinical period of RA during which anti-cyclic citrullinated protein can be detected without symptoms of arthritis [42]. Finally, due to the difficulty of translating the high dose of tolerogenic DC used in mice to an equivalent dose for treating patients, it is remarkable that the single dose used in this work was sufficient to induce protection. Although the main application of DC immunotherapy has been in cancer research, in a recent work the generation and characterization of clinical-grade human tolerogenic DC to be used in RA has been reported [43].

So far, we have not identified yet the molecular nature of the TE component/s responsible for the modulation of TLR-induced DC maturation. However, unpublished data from our lab have demonstrated that a low molecular weight fraction from TE was capable of down-modulating TLR-induced DC maturation by reducing the production of pro-inflammatory cytokines. Related to this, experiments attempting to identify this fraction are currently being performed.

In conclusion, we demonstrate that the combination of helmint Ags together with a TLR ligand can induce tolerogenic DC which are able to prevent CIA symptoms. These findings open up new possibilities to design DC-based therapy against exacerbate inflammatory responses.

## Supporting Information

Figure S1 **Cells from DLN of differentially-treated DC recipient mice were stimulated with PMA, ionomycin and brefeldin A for 5 hr and stained with fluorescent antibodies for CD4, Foxp3, B220 and F4/80 and intracellular IL-10.** A) Plots show the percentage of CD4+Foxp3+ (upper panel) and histogram for the IL-10+ in CD4+Foxp3+ and CD4+Foxp3- cells (lower panel), B) Plots show the percentage B220+IL-10+ cells. C) Plots show the percentage F4/80+IL-10+ cells. Data are representative of two independent experiments.
(TIF)

Figure S2 **Cells from DLN of CII pulsed T/C-DC recipient mice were sorted by using flow cytometer FACSAriaTM II on day 7 of onset.** A) CD4+CD25- and CD4+CD25+ cells populations were sorted with more than 96% of purity. B) The percentage of Foxp3+ cells in CD4+CD25+ and CD4+CD25- cells population is shown.
(TIF)
