# Supplementary figures and images for: Helminth Antigens Enable CpG-Activated Dendritic Cells to Inhibit the Symptoms of Collagen-induced Arthritis through Foxp3+ Regulatory T Cells
Source: PLoS One. 2012 Jul 25;7(7):e40356. doi: 10.1371/journal.pone.0040356 (PMC3405066; doi:10.1371/journal.pone.0040356)

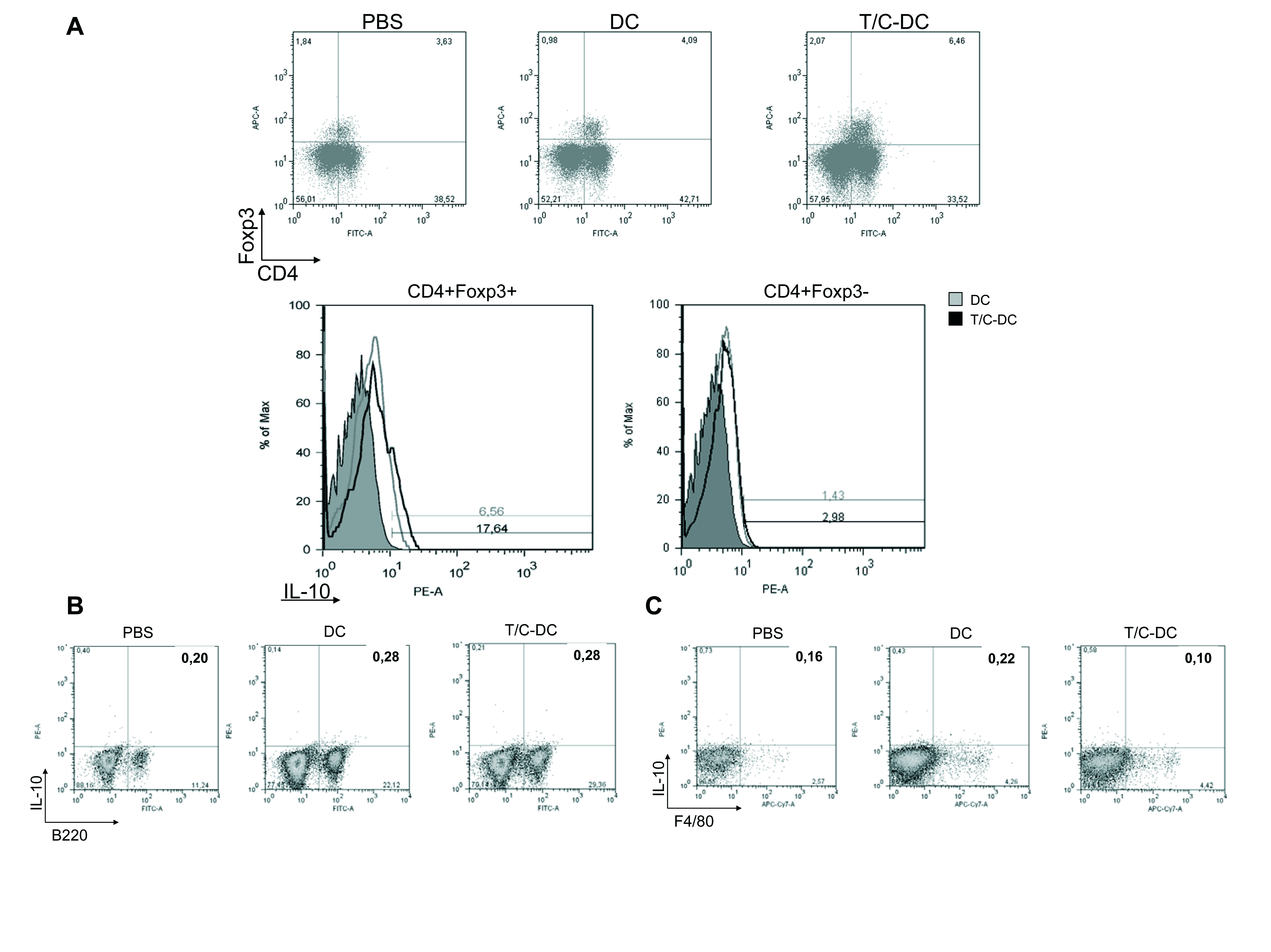

Supplement: Figure S1 — Cells from DLN of differentially-treated DC recipient mice were stimulated with PMA, ionomycin and brefeldin A for 5 hr and stained with fluorescent antibodies for CD4, Foxp3, B220 and F4/80 and intracellular IL-10. A) Plots show the percentage of CD4+Foxp3+ (upper panel) and histogram for the IL-10+ in CD4+Foxp3+ and CD4+Foxp3- cells (lower panel), B) Plots show the percentage B220+IL-10+ cells. C) Plots show the percentage F4/80+IL-10+ cells. Data are representative of two independent experiments. (TIF) [file pone.0040356.s001.tif]

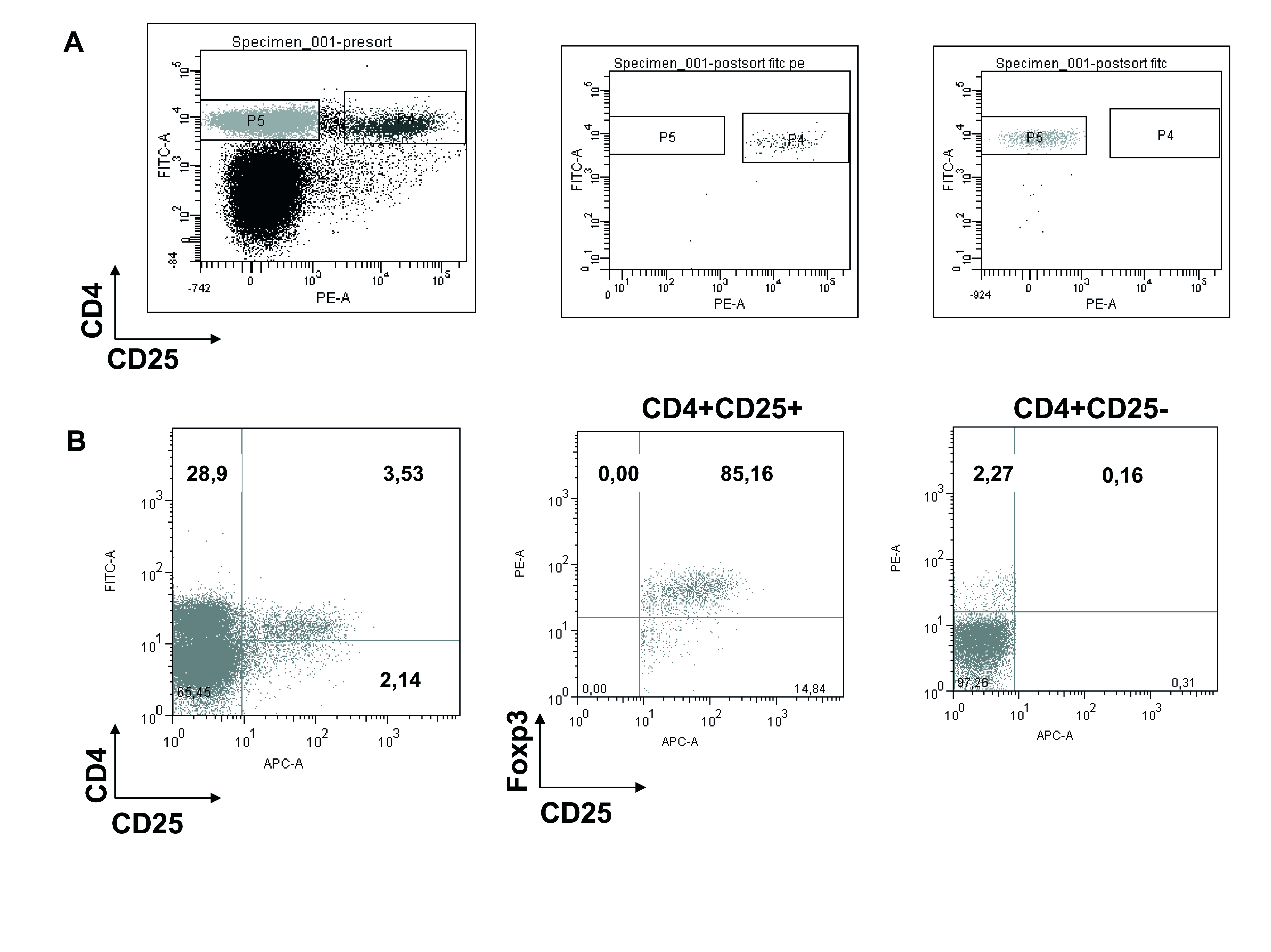

Supplement: Figure S2 — Cells from DLN of CII pulsed T/C-DC recipient mice were sorted by using flow cytometer FACSAriaTM II on day 7 of onset. A) CD4+CD25- and CD4+CD25+ cells populations were sorted with more than 96% of purity. B) The percentage of Foxp3+ cells in CD4+CD25+ and CD4+CD25- cells population is shown. (TIF) [file pone.0040356.s002.tif]
